# Posterior reversible encephalopathy syndrome secondary to asymptomatic poststreptococcal glomerulonephritis in a child with sickle cell anemia: a case report

**DOI:** 10.1186/s13256-017-1559-x

**Published:** 2018-02-01

**Authors:** Ehab Hanafy, Duaa Alshareef, Suhaila Osman, Abdullah Al Jabri, Faisal Nazim, Gihan Mahmoud

**Affiliations:** 1 Prince Sultan Oncology Center, King Salman Armed Forces Hospital, Tabuk, 100 Kingdom of Saudi Arabia; 2 Pediatric Department, King Salman Armed Forces Hospital, Tabuk, Kingdom of Saudi Arabia

**Keywords:** Sickle cell anemia, Posterior reversible encephalopathy syndrome, Stroke, Hypertension, Glomerulonephritis

## Abstract

**Background:**

Posterior reversible encephalopathy syndrome is a neurotoxic condition that occurs as a result of the failure of posterior circulatory autoregulation in response to acute changes in blood pressure. Overperfusion with resultant disruption of the blood-brain barrier results in vasogenic edema, but not infarction. Posterior reversible encephalopathy syndrome can be the presenting feature of postinfectious glomerulonephritis, which has been reported in approximately 5% of hospitalized children, and it has been reported in very few cases of adult patients with sickle cell anemia. We report a very rare case of posterior reversible encephalopathy syndrome that occurred in a child with sickle cell anemia. This presentation should be differentiated from other neurologic manifestations that occur in patients with sickle cell anemia, because management is totally different.

**Case presentation:**

We report what is to our knowledge the first reported case of a 9-year-old Saudi girl with sickle cell anemia who developed posterior reversible encephalopathy syndrome secondary to asymptomatic poststreptococcal glomerulonephritis. This occurred after full recovery from acute chest syndrome and severe vaso-occlusive crisis.

**Conclusions:**

The purpose of this report is to emphasize that all efforts should be made to explore the causes of different neurologic manifestations that occur in patients with sickle cell anemia, because this will require different pathways of management.

## Background

Stroke in sickle cell anemia (SCA) is a serious complication that occurs in various forms and needs to be managed promptly and effectively to prevent any irreversible neurologic deficit. This neurologic complication seen in children with SCA is different from a rare neurologic disorder known as *posterior reversible encephalopathy syndrome* (PRES), which is a clinicoradiological entity characterized by rapid onset of an altered level of consciousness, visual disturbance, headache, and seizures.

The exact pathophysiological mechanisms that could explain PRES are debatable; however, the most popular one is related to increasing blood pressure causing failure of brain autoregulation. Uncontrolled hypertension leads to hyperperfusion and cerebral vessel damage, resulting in interstitial extravasation of proteins and fluids, causing vasogenic edema.

The other less popular theory postulates that PRES is due to vascular endothelial dysfunction that may occur as a result of systemic inflammatory conditions. This can be explained by the presence of a direct association of PRES with systemic inflammatory processes such as sepsis, eclampsia, transplant, and autoimmune disease [1]. We present a rare case of PRES in a child with SCA, and because it is a sort of neurologic insult, it should be cautiously differentiated from other neurologic manifestations that occur in SCA in which the management pathway is different.

## Case presentation

A 9-year-old Saudi girl was presented to our hospital with a fever of 38.5 °C, cough, and shortness of breath. Her oxygen saturation was 88% on room air; her blood pressure was normal for her age (110/75 mmHg); and she had no tachycardia (80 beats/minute). She had had abdominal pain for 2 days that was not responding to analgesics. She had been diagnosed with SCA at the age of 2 years. Both her parents are consanguineous and have the sickle cell trait, whereas all her siblings are sickle cell-negative on the basis of routine screening. The patient had no history of hypertension, diabetes, or other conditions of medical concern; no history of previous surgeries; and no history of drug allergies. Her developmental history was compatible with her age, and her vaccinations were up-to-date. She was followed regularly at our pediatric hematology clinic and receiving regular hydroxyurea and folic acid supplements.

On examination, the patient had no organomegaly; her ear, nose, and throat examination was unremarkable; and her initial neurologic examination was unremarkable with normal motor power, sensations, and reflexes. Subsequently, she was admitted to the hospital and diagnosed with acute chest syndrome on the basis of her clinical manifestations and bilateral lung infiltrates seen on chest x-rays. Her concomitant abdominal pain was attributed to vaso-occlusive crisis. She was started on full supportive care, including antibiotics, analgesics, intravenous fluids, and oxygen supplementation, and she was given packed red blood cells once with minimal improvement. She underwent exchange transfusion because of progressive respiratory distress and increased oxygen requirement.

During her stay at the pediatric intensive care unit and after 5 days of exchange transfusion, the patient’s respiratory distress and radiologic evidence of acute chest syndrome resolved; however, she developed systemic hypertension and was started on antihypertensive medications with slight improvement. Echocardiography showed mild mitral regurgitation with dilation of the left ventricle, and Doppler ultrasound showed patent hepatic and renal vasculature. The results of her blood workup were within normal ranges, apart from creatinine, which was slightly elevated (Table 1).

**Table 1:** Results of blood workup at time of initial hypertensive episode

	Value	Normal range
Sodium	136	132–146 mmol/L
Potassium	4.9	3.6–5.0 mmol/L
Chloride	109	98–107 mmol/L
Enzymatic bicarbonate	18	22–29 mmol/L
Urea nitrogen	4.92	1.6–4.6 mmol/L
Creatinine	109	20–70 μmol/L
Total protein	65	64–86 g/L
Albumin	28	38–56 g/L
ALP	144	218–499 U/L
AST (SGOT)	46	15–37 U/L
ALT (SGPT)	37	24–49 U/L
Total bilirubin	8	0–14 μmol/L
WBC	21.98	5.0–13.0 × 10^3^/μl
Hemoglobin	8.4	11.5–14 g/dl
PLT	113	180–400 × 10^3^/μl

*Abbreviations: ALP* Alkaline phosphatase, *ALT* Alanine aminotransferase, *AST* Aspartate aminotransferase, *PLT* Platelets, *SGOT* Serum glutamic-oxaloacetic transaminase, *SGPT* Serum glutamic-pyruvic transaminase, *WBC* White blood cell count

Four days later, the child had marked elevation of her blood pressure that was followed by seizures and sudden loss of vision. On examination, she was drowsy, had a Glasgow Coma Scale score of 10 on a scale of 15, and was responding to verbal stimuli. Her neurologic examination showed squinting of the right eye to the right side, and her pupils were equal and reactive to light. She was unable to recognize any objects. She had no signs of meningeal irritation, and results of her motor and sensory examinations were normal. Examination of her chest revealed that it was clear, and her liver was palpable 4 cm below the costal margin. Review of her other systems was unremarkable.

**Fig. 1:**
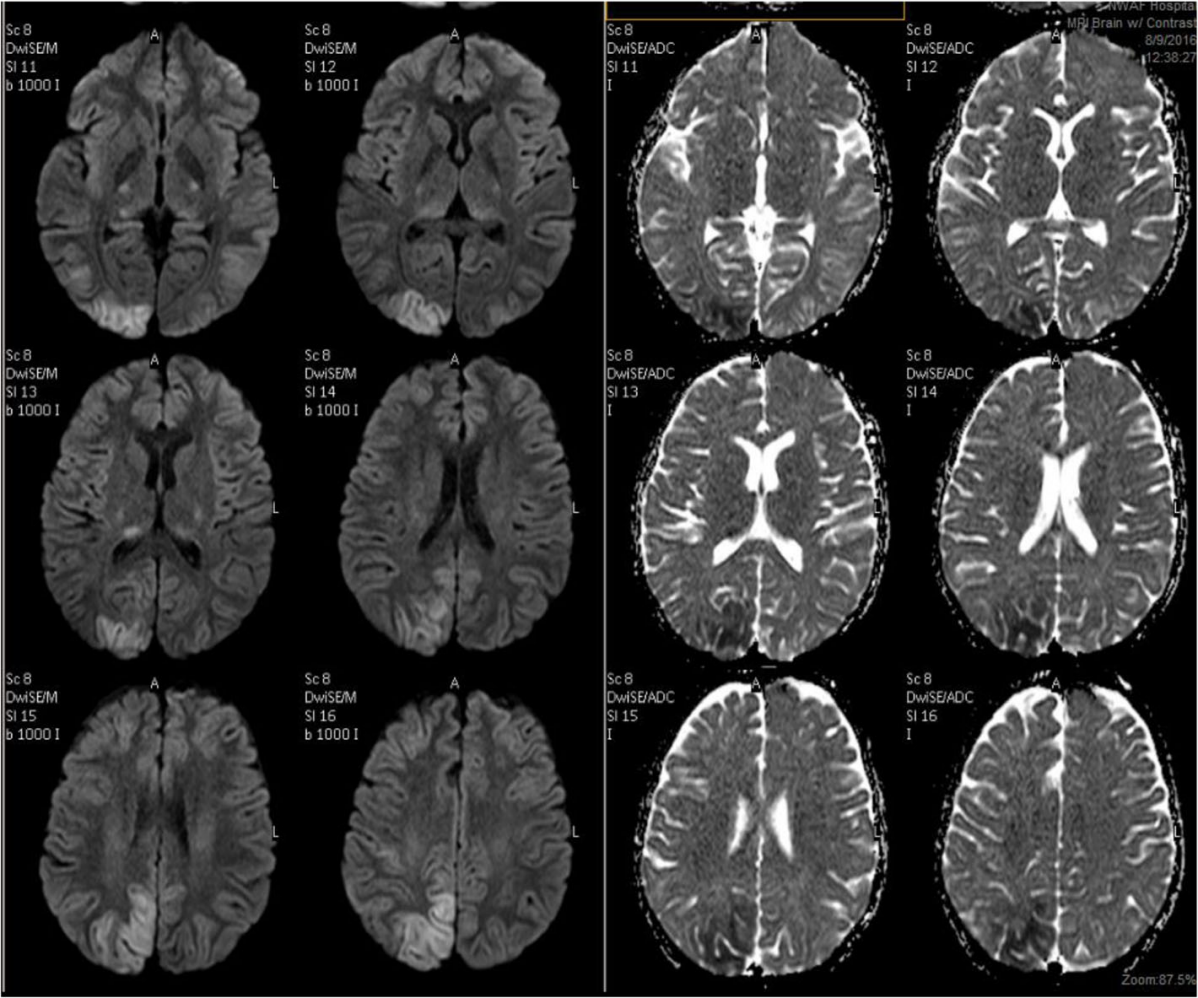
Brain magnetic resonance imaging scans showing tiny areas of recent infarctions with a picture diagnostic of posterior reversible encephalopathy syndrome

Urgent magnetic resonance imaging (MRI) of the patient’s brain (Fig. 1) showed an ill-defined, nonenhanced area of abnormal hyperintense signaling on fluid-attenuated inversion recovery (FLAIR) T2-weighted (T2W) images and isointensity on T1-weighted (T1W) images with restricted diffusion. A low apparent diffusion coefficient value was seen involving the cortex of the right occipitoparietal lobe and a tiny area of the same signal intensity within the right thalamic region suggestive of recent infarctions. The imaging studies also showed multiple ill-defined, nonenhanced patches of abnormal hyperintense signaling on FLAIR images, as well as isointense signaling on T1W images with no restriction of diffusion seen involving the cortex of the occipitoparietal region bilaterally, suggesting posterior reversible hypertensive encephalopathy. The patient’s electroencephalogram was reported as normal. She was kept on full supportive measures, including antihypertensive and antiepileptic medications as well as antibiotics, in addition to intravenous fluids to improve renal function.

Further investigations were done to rule out a renal cause of hypertension. This included measurement of renin and aldosterone levels, which were normal. Her anti-streptolysin O titer was high at 490 IU/ml (normal range 0–200 IU/ml), and her C3 level was significantly low at 0.22 g/L (normal range 0.9–1.8 g/L). Urine analysis revealed 25–50 red blood cells (Table 2).

**Table 2:** Urine analysis

Analysis	Reference	Value
SG	1.010–1.025	1.015
pH	6.0–7.0	7.0
Leukocyte	Negative	Negative
Nitrite	Negative	Negative
Protein	Negative	30 mg/dl
Glucose	Negative	Negative
Ketone	Negative	Trace
Urobilinogen	0.2–1 mg/dl	1.0 mg/dl
Bilirubin	Negative	Negative
Red blood cells	Negative	Large
Urine Appearance		Bloody
Urine White blood cells		Negative
Urine Red blood cells		25–50
Urine Bacteria		Negative
Urine RBC casts		0–2

*RBC* Red blood cell, *SG* Specific gravity

The child started to improve gradually over a 1-week period with full restoration of vision and adequate seizure control. Her general condition improved markedly, with an unremarkable systemic examination just within 10 days of her abrupt clinical deterioration.

Our impression was that our patient had PRES secondary to postinfectious glomerulonephritis. Recent brain infarctions can follow a severe attack of acute chest syndrome and vaso-occlusive crisis. Our patient is currently on a regular blood transfusion program as a secondary prevention of stroke. Brain MRI was repeated 2 months after presentation, and it showed that the previously mentioned scattered high T2W and high FLAIR signal intensity of the cortex and subcortical white matter with restricted diffusion were almost completely resolved. The results of repeat anti-streptolysin O titer, C3 level, and urine analysis were completely normal. Our patient is currently in good condition with no neurologic manifestations, and she attends regular monthly follow-up visits at our hematology clinic.

## Discussion

We present a rare case of PRES in a child with SCA that was mostly secondary to glomerulonephritis. PRES as a neurologic condition should be interpreted carefully in patients with SCA, because other neurologic conditions that occur in SCA have different pathways of management.

Blood pressure is generally lower than normal in patients with SCA. Those with higher levels (relative to this population) have an increased risk of stroke and death [2].

It has been shown that, compared with patients with SCA with lower blood pressure, patients with SCA and blood pressure values in the high range of normal have higher serum creatinine levels and an increased risk of pulmonary hypertension [3, 4]. It is possible, though unproven, that moderate degrees of hypertension are more damaging to the kidney, particularly in patients with SCA compared with those without SCA. Therefore, it is our practice to treat these patients aggressively to avoid any possible renal impairment.

Hypertension is seen in only 2–6% of patients with SCA [5]. This has been attributed to several factors, including sodium and water wasting as a result of pathological involvement of the renal medulla, compensatory systemic vasodilation due to significant reductions in the microcirculation, and increased levels of serum prostaglandins and nitric oxide.

SCA can cause different renal syndromes and diseases that reflect the complex vasculopathy of SCA and the propensity of red blood cells to sickle in the renal medulla because of its hypoxic, acidotic, and hyperosmolar conditions. This includes glomerulopathies, chronic kidney disease, acute kidney injury, impaired urinary concentrating ability, hematuria, and other rare renal conditions. Renal involvement contributes substantially to the diminished life expectancy of patients with SCA, accounting for 16–18% of mortality [6].

It is of crucial importance to differentiate those renal conditions that occur as a result of vasculopathy in SCA and other renal diseases that are not related, because this will require pursuing a different management approach. Strokes in children and young adults with SCA continue to be a major cause of morbidity. Understanding the epidemiology and pathophysiology of overt strokes is critical to immediate and multidisciplinary team management. This was initially described in 1923 in a case of a child with SCA who had seizures and acute left hemiparesis [7]. In 1970, Powars *et al*. [8] described for the first time the high rate of overt ischemic strokes in children and young adults with SCA (6%), as well as their high recurrence rates in the first 2 years after the initial event (50%) and in the following 9 years poststroke (66%). In 1990, investigators in the Cooperative Study of Sickle Cell Disease provided the most definitive and comprehensive study of the natural history of strokes across the life-span [9].

Children and adults with SCA have a high prevalence (4.01%) and incidence (0.61 per 100 patient-years) of cerebrovascular accidents [9]. In patients with SCA, ischemic strokes were observed to have a bimodal distribution. They are more common in children and older adults. The lowest incidence was reported in adults aged 20–29 years. The risk factors associated with ischemic strokes include transient ischemic attack, low steady-state hemoglobin concentration, recent episode of acute chest syndrome, and elevated systolic blood pressure. In individuals with SCA, hemorrhagic stroke was most frequent in the 20- to 29-year-old age group. The associated risk factors included low steady-state hemoglobin and high leukocyte count [9].

PRES usually presents with rapid onset of symptoms, including headache, seizures, altered level of consciousness, and visual disturbance. The disorder should be associated with an acute episode of hypertension [10, 11].

Chronic kidney disease and acute kidney injury are both commonly present in patients with PRES [12]. The disorder is commonly associated with conditions that coexist in patients with renal disease, such as hypertension, vascular and autoimmune diseases, exposure to immunosuppressive drugs, and organ transplant.

PRES can be the presenting feature of postinfectious glomerulonephritis, which was reported in approximately 5% of hospitalized children. In these patients, hypertension is usually severe and is accompanied by headache, vomiting, depressed sensorium, confusion, visual disturbances, aphasia, memory loss, coma, and convulsions. The mechanism of hypertension is most likely retention of sodium and water with resulting expansion of the extracellular space [13].

Our patient had no suspected history of streptococcal infection or gross hematuria; however, the possibility of poststreptococcal glomerulonephritis in children with symptoms that may be secondary to hypertension even in the absence of gross hematuria or a history of a preceding streptococcal infection should always be considered.

PRES has been reported immediately after blood transfusion in a small number of patients. This is usually attributed to acute volume overloads by transfusion exceeding the capacity of autoregulation of perfusion pressure, resulting in vasogenic edema [14]. However, this was not reported after exchange transfusion.

The severity of clinical symptoms varies from one patient to another. The visual disturbance may present as blurring of vision, homonymous hemianopsia, or even cortical blindness [11]. Patients may be slightly confused or agitated or may become comatose [15]. Other symptoms that are less commonly seen include nausea, vomiting, and brain stem deficits [11, 15]. Seizures and status epilepticus are common, and nonconvulsive status epilepticus may be more frequent than generalized status epilepticus [16]. Nonconvulsive status epilepticus should be suspected in patients with prolonged states of altered consciousness and may be mistaken for postictal confusion. Signs of nonconvulsive seizures may include stereotypic movements such as staring, eye blinking, or head turning. Postictal confusion usually lasts for hours. Both PRES and nonconvulsive status may persist for several days and may be mistaken for psychosis, drug intoxication, or psychogenic states [17].

PRES in adult patients with SCA has been reported in association with severe acute chest syndrome, recurrent priapism, sudden elevation of blood pressure, or renal failure. In all these instances, PRES was attributed to abrupt elevation in blood pressure or blood volume [18, 19].

The pathophysiology of PRES remains controversial. The two main hypotheses contradict each other. One involves impaired cerebral autoregulation with subsequent increase in cerebral blood flow, whereas the other theory involves endothelial dysfunction with cerebral hypoperfusion. This hypoperfusion hypothesis is more applicable in cases of PRES associated with cytotoxic therapy. In both hypotheses, cerebral blood perfusion abnormalities are related to blood-brain barrier dysfunction with cerebral vasogenic edema [20].

The most characteristic imaging pattern in PRES is the presence of edema involving the white matter of the posterior portions of both cerebral hemispheres, especially in the parieto-occipital regions. This is usually reported in a relatively symmetric pattern with sparing of the calcarine and paramedian parts of the occipital lobes [15]. However, other structures (such as the brain stem, cerebellum, and frontal and temporal lobes) may also be involved. The abnormality affects primarily the subcortical white matter; yet, the cortex and the basal ganglia may also be involved [21]. Gyriform signal enhancements are rare. Furthermore, parenchymal hemorrhage can occur in complicated cases.

In many cases, distinguishing acute cerebral infarcts from PRES is still challenging [22]. MRI is the imaging modality of choice, and although both PRES and cerebral infarcts present with abnormal hyperintensities on fluid-sensitive sequences, the distribution of signal abnormalities is usually different in both disorders. Diffusion-weighted imaging (DWI) typically is positive in cerebral infarcts, whereas DWI in PRES may or may not be positive [23, 24]. Performing diffusion-weighted MRI of the brain is important to rule out the diagnosis of PRES.

No clinical trials have evaluated the management of PRES, but rapid withdrawal of the trigger factor appears to improve the rate of recovery and avoids possible complications. The prognosis is good because complete reversal of clinical symptoms usually occurs within a few days. Delay in diagnosis and treatment may lead to serious and permanent neurologic sequelae. Therefore, increased awareness of PRES is of crucial importance for the pediatrician.

## Conclusions

Unlike stroke, PRES *per se* is not an indication for a blood transfusion program in patients with SCA. However, when neurologic symptoms occur, further investigations should be carried out to verify whether these neurologic manifestations are directly related to SCA or whether other pathology is involved, because this will call for extremely different management pathways.

## Abbreviations

ALP: Alkaline phosphatase
ALT: Alanine aminotransferase
AST: Aspartate aminotransferase
DWI: Diffusion-weighted imaging
FLAIR: Fluid-attenuated inversion recovery
MRI: Magnetic resonance imaging
PLT: Platelets
PRES: Posterior reversible encephalopathy syndrome
RBC: Red blood cells
SCA: Sickle cell anemia
SG: Specific gravity
SGOT: Serum glutamic-oxaloacetic transaminase
SGPT: Serum glutamic-pyruvic transaminase
T1W: T1-weighted
T2W: T2-weighted
WBC: White blood cell count
